# Lancing the Gums

**Published:** 1871-03

**Authors:** 


### LANCING THE GUMS.

Dr. E. Bentley, U. S. A., says: Lancing the gums is a process which is becoming too antiquated for the present generation. I know of physicians who have been five or ten years in practice, and have never scarified the gums of a teething child. It is probable that very few who practiced it when it was in common use, and who had a fair opportunity of observing its great utility in many instances, have abandoned it. The desuetude is an innovation not founded on experience. It attaches mostly to the younger members of the profession who have grown up under the influence of the popular prejudice against bleeding, mercury, and "mineral poisons," engendered by homeopaths, hydropaths, eclectics, botanics, magnetizers, spiritists, and charlatans and buffoons of a thousand species.

It is easy to make out a case against the gum-lancet when parents are in the seat of judgment: "The poor, dear child;" "the inflamed and tender gums;" "the danger of bleeding to death;" that "horrid lancet:"—such arguments soon settle the question. Policy often goes further than conscience with the physician. He will lose favor by enforcing his judgment. Hence he yields to the situation, and at length conforms his creed to his practice.

There are three objections to scarifying the gums: First, the pain and struggling of the child; second, the increased difficulty of teething arising from the cicatrix; third, the danger of hemorrhage.

As for the pain, it is trifling, and unworthy of notice.

The consequent relief is much more than sufficient to counterbalance the pain. Often the itching of the gums is so intolerable that the impression of the lancet is agreeable. I have known a child to close its jaws on the instrument and press it into the gums with evident satisfaction.

The struggling of the child, and its fright, are of greater importance, especially if the operation be bunglingly done, as is often the case. There is but one right way of doing it. Take your seat behind the child as it rests on the nurse's lap in a proper light, and placing your knees toward its back, draw its head down between your knees. Let the nurse hold the infant's hands. What with your knees and your two hands, the head is now completely under your control. Grasp it between your two palms, and as it opens its mouth to cry, thrust one or two fingers of the left hand in its mouth to keep the jaws apart, and use the lancet with the other hand. By this method you have the most perfect command of the head, and can cut exactly in the spot and to the extent you desire. I am thus precise in the description because I have so often seen the operation so awkwardly undertaken as to fail of its purpose, and to endanger serious wounding of the child's mouth.

Some writers have recommended cutting down on the outside of the gum, toward the root of the tooth, and not on the ridge, in a perpendicular direction, toward the crown. If the gum be much swollen, and the tooth deep, this plan may answer.

In some cases it is sufficient simply to relieve the distension by scarifying, without cutting down to the teeth. The loss of a few drops of blood in this way is often eminently useful, aside from any topical effect.

The second objection, viz : the cicatrix, is scarcely worth a serious refutation. When we consider that the tooth effects a passage by inducing absorption of the gum through pressure, it is evident that absorption will be more easily accomplished where there is a cicatrix than where the tissue possesses all its original vitality and power of resistance. Repeated incisions, therefore, have an effect opposite to that which the popular mind ascribes to them. By weakening the vitality of the tissue they facilitate the exit of the tooth.

Besides, the idea of induration, as attached to the cicatrix, is probably fallacious. I have never observed any induration of the gums, after scarification—perhaps because they heal so speedily, and are kept constantly moist.

Finally, we come to the most important objection—the danger of hemorrhage. This is of rare occurrence. In an experience of more than forty years, during which it has always been my practice to use the lancet freely in dentition, not a single instance has occurred to me. I have heard the same testimony from my father, after forty years of practice, in which he never hesitated to lance the gums of a teething child.

Nearly forty years ago, this subject was canvassed in the Medical Society of Wilmington, Delaware—of which I was then a junior member. My recollection of that discussion is distinct. Eight or ten physicians were present, some of whom had been many years in service. The practice of cutting the gums was then universal. And yet, not a single case of fatal or dangerous hemorrhage, from lancing the gums, had occurred to any one of them, and only one case had come within their knowledge. A child named Collins, the patient of a physician then deceased, had been visited, in consultation, by several of those present, and died after a number of days of hemorrhage.

But the experience of others is not uniformly of the same tenor. My friend, Dr. Hatch, of Sacramento, in a paper read before the Medical Association of that city, mentions four cases of hemorrhage following incision of the gums, which have come to his knowledge—all of which proved fatal. In these cases, however, there was pre-existing disease which, in all probability, would have destroyed life, had the gums been left intact. Further, they had been treated with calomel, until the peculiar effect of that agent on the blood appeared to be fully established. Dr. Hatch infers that the operation should never be performed on anemic children, or on those whose appearance might lead to a suspicion of the hemorrhagic tendency; and that it should be particularly avoided in patients under the influence of mercury.

The experience of Dr. Hatch is exceptional, and not to be accepted as a guide, in regard to the frequency of hemorrhage from this cause. It is extraordinary that so many cases should have fallen under the observation of a single practitioner. There have been deaths from hemorrhage resulting from the extraction of teeth—perhaps as large a proportion as from cutting the gums. The same may be said of many other minor operations But such extraordinary accidents are not allowed to deter us from operating, when the occasion presents. I therefore conclude that the irritation of the gums from teething is so much more dangerous, under all circumstances, than the cutting of them with the lancet, as to justify the operation, without regard to consequences.

